# Waist-to-height-ratio is associated with sustained hypertension in children and adolescents with high office blood pressure

**DOI:** 10.3389/fcvm.2022.1026606

**Published:** 2023-01-11

**Authors:** Norrarath Nimkarn, Anyamanee Sewarit, Kwanchai Pirojsakul, Witchuri Paksi, Songkiat Chantarogh, Pawaree Saisawat, Kanchana Tangnararatchakit

**Affiliations:** Department of Pediatrics, Faculty of Medicine Ramathibodi Hospital, Mahidol University, Bangkok, Thailand

**Keywords:** waist-to-height-ratio, body mass index, ambulatory blood pressure monitoring, sustained hypertension, children, adolescents

## Abstract

**Background:**

Waist-to-height-ratio (WHtR) has been proposed as another indicator for cardiometabolic risk factors including hypertension. Normally, hypertension can be diagnosed in the office setting by detecting high blood pressure for three occasions. However, patients with high office blood pressure may not exhibit high blood pressure outside the office. Ambulatory blood pressure monitoring (ABPM) is a procedure to measure blood pressure over 24-h. Sustained hypertension is characterized as hypertension detected by both office measurement and ABPM. This study aimed to evaluate the performance of WHtR in the diagnosis of sustained hypertension in patients with high office blood pressure.

**Materials and methods:**

Demographic data, height, body weight, body mass index (BMI), and waist circumference were retrospectively reviewed in children and adolescents who underwent ABPM due to persistently high office blood pressure. Patients were separated into two groups: a sustained hypertension group and a normal ABPM group. BMI was adjusted to z-score using the WHO Anthroplus software. WHtR was calculated by the formula: waist circumference (cm)/height (m). The performances of different parameters were analyzed using the receiver operating characteristic (ROC) curve and multivariate logistic regression.

**Results:**

Sixty patients (63% male) with a mean age of 12.9 ± 3.7 years had persistently high office blood pressure. Twenty-nine (48.3%) had high ambulatory blood pressure parameters so-called “sustained hypertension.” The sustained hypertension group had a higher mean BMI z-score (2.32 vs. 1.31, *p* = 0.01) and a higher mean WHtR (57.7 vs. 49.2 cm/m, *p* < 0.001) than those of the normal ABPM group. For the diagnosis of sustained hypertension, the ROC analysis revealed that WHtR had a greater area under the ROC curve (AUC) than that of BMI z-score (0.772 vs. 0.723). WHtR remained associated with sustained hypertension (OR 1.2, 95% CI 1.022–1.408, *p* = 0.026) after adjusting for age, gender, and BMI z-score.

**Conclusions:**

Apart from being a more user-friendly metric, WHtR tended to outperform BMI z-score in predicting sustained hypertension in children and adolescents with persistently high office blood pressure.

## 1. Introduction

The increased prevalence of hypertension in children and adolescents has become a major public health issue (1, 2). Several studies have found that high blood pressure in childhood increased the likelihood of adult hypertension, which is a major contributor to cardiovascular disease later in life (3, 4). A report from the Thai National Health Examination Survey (5) showed that 9.4% of adolescents aged 10–19 years had high blood pressure and this prevalence was higher than those of the recent national surveys from South Korea and Canada (6, 7). Obesity was found to be an important determinant to high blood pressure seen in Thai school-aged children. It was revealed that obesity was significantly associated with high blood pressure and it increased the risk of pre-hypertension and hypertension by 9 and 10.6-fold, respectively (8, 9).

Systematic reviews showed an association between hypertension and body mass index (BMI) together with various measures of abdominal adiposity and the hypertension rates increased in a graded manner as adiposity increased (10–14). Generally, adiposity indicator such as BMI is correlated with hypertension (15–18). Meanwhile, abdominal obesity has also been recognized as a risk factor for hypertension in children and adolescents by using different measuring methods and various indices such as waist circumference (WC) and waist-to-height-ratio (WHtR) (18–24). WHtR can be calculated by dividing WC by height to represent an individual's size. WHtR varies only slightly across age and gender, therefore it does not need to be expressed as a z-score as does BMI (25). Some studies in children even suggested that WHtR was more strongly linked to high blood pressure than was BMI (26–28), while the others indicated that WHtR had a weaker relation to blood pressure compared to that of BMI (29, 30). As a result, the performances of these parameters for predicting hypertension in children and adolescents remain unclear.

Normally, hypertension in children and adolescents can be diagnosed in the office setting by detecting blood pressure greater than the 95th percentile for gender, age, and height for three occasions. However, some children and adolescents may have high blood pressure in the office but do not show high blood pressure outside the office. In 2017, the American Academy of Pediatrics clinical practice guidelines recommended that ambulatory blood pressure monitoring (ABPM) be used to confirm hypertension in children and adolescents who have persistently high office blood pressure for three occasions (3). ABPM is a procedure in which blood pressure is measured every 20–30 min over 24-h using a portable device. Those with high blood pressure for both office measurement and ABPM are called having “sustained hypertension.” Previous studies on the relationship between BMI, WHtR, and the diagnosis of hypertension in children and adolescents typically defined hypertension mainly by using office blood pressure measurement with only a few studies employing ABPM in children and adolescents with obesity (31–33). The present study aimed to evaluate the performances of BMI z-score and WHtR in the diagnosis of sustained hypertension detected by ABPM in children and adolescents with high office blood pressure.

## 2. Materials and methods

### 2.1. Participating patients

Patients aged ≥6 years referred to the pediatric hypertension clinic at Ramathibodi Hospital Mahidol University due to high office blood pressure on three occasions and subsequently underwent 24-h ABPM were enrolled in the present study. Exclusion criteria included patients who did not have sufficient ABPM data, were previously diagnosed with hypertension, and had any underlying diseases or received any medications that may affect blood pressure.

### 2.2. Anthropometric data

Demographic data, height, body weight, BMI, and waist circumference were collected. Waist circumference was measured while standing straight using a measurement tape with a precision of 1 mm. The tape was placed at the midline between the bottom of the lowest rib and the iliac crest. WHtR was calculated by the following formula: waist circumference (cm)/height (m) and BMI was adjusted to z-score for age and gender using the World Health Organization (WHO) Anthroplus software (34). Obesity was defined as a BMI z-score > 2.

### 2.3. Blood pressure measurement

Office blood pressure was measured with an oscillometric device two times in the right arm while seated, using standard blood pressure measurement practice and appropriate cuff size. An average of the two blood pressure values was considered to be a blood pressure value for each occasion. High office blood pressure is defined as a systolic blood pressure (SBP) or a diastolic blood pressure (DBP) ≥95th percentile for gender, age, and height in children aged < 13 years; or ≥ 130/80 mmHg in children aged ≥ 13 years for three occasions according to the current pediatric guidelines (3). For office blood pressure, mean blood pressure was an average of the blood pressure values from three occasions while maximum blood pressure was the highest blood pressure value among three occasions.

ABPM was performed using a TM-2430 (A&D, Japan) device, which has been validated for use in pediatric patients (35). An appropriate cuff for each patient was applied on the non-dominant arm by the trained healthcare provider. The device was set to record blood pressure every 20 min during awake and every 30 min during sleep for a period of 24-h. Patients were instructed to continue their normal daily activities, avoid strenuous activities, and record their activities including the actual sleep and awake periods. In each patient, ABPM data were considered sufficient if there were ≥40 valid blood pressure readings for the entire 24-h period. Hypertension by ABPM is defined as a mean SBP or a mean DBP ≥ 95th percentile for gender and height, and SBP or DBP load ≥25% for either awake or asleep or both periods while prehypertension is defined as a mean SBP or a mean DBP < 95th percentile for gender and height, but SBP or DBP load ≥25% for either awake or asleep or both periods according to the guidelines by the American Heart Association (36). Based on the results of ABPM, patients were separated into two groups: a sustained hypertension group and a normal ABPM group. The normal ABPM group included patients with prehypertension and white coat hypertension.

To compare blood pressure parameters between patients of different ages, genders, and heights, blood pressure parameters were converted to blood pressure indices with the following formula: blood pressure value/cut-off value for high blood pressure for each patient.

### 2.4. Statistical analysis

Statistical analysis was performed using IBM SPSS^®^ Software, Version 26. The distribution of each parameter was tested with the Kolmogorov-Smirnov test. Descriptive data were presented as number (percentage), mean ± standard deviation (SD), or median (interquartile range, IQR) as appropriate. Demographic data were compared between the sustained hypertension group and the normal ABPM group. For comparative analysis, the chi-square test or Fisher's exact test was used for categorical data; and the Student *t*-test or Mann–Whitney *U*-test was used for continuous data, as appropriate. The receiver operating characteristic (ROC) curve was used to analyze the performances of the BMI z-score and WHtR for the diagnosis of sustained hypertension. Univariate logistic regression analysis was used to test the parameters associated with sustained hypertension. The parameters that were significantly associated with sustained hypertension from the univariate analysis, were further added to the multivariate logistic regression model. A *p* ≤ 0.05 was defined as statistical significance.

## 3. Results

### 3.1. Patient characteristics

A total of 72 patients with persistently high office blood pressure were enrolled in the present study. Twelve patients with congenital anomalies of the kidney and urinary tract, attention deficit hyperactivity disorder, systemic lupus erythematosus, vesicoureteral reflux, obstructive sleep apnea, and coarctation of aorta were excluded as their underlying diseases or medication uses might affect blood pressure. Among sixty patients (38 males) with a mean age of 12.9 years, 29 patients (48.3%) had sustained hypertension. The demographic and clinical data between the two groups are shown in Table 1. A higher mean BMI z-score (2.32 ± 1.51 vs. 1.31 ± 1.49, *p* = 0.01) and a more proportion of obesity [20 (69%) vs 10 (32%), *p* = 0.04] were detected in the sustained hypertension group compared with the normal ABPM group. The sustained hypertension group also had a substantially higher mean waist circumference (86.7 ± 17.8 vs. 77.7 ± 16.7, *p* = 0.048) and a higher mean WHtR (57.7 ± 8.5 vs. 49.2 ± 9.2, *p* < 0.001*)* than those of the normal ABPM group.

**Table 1:** Demographic and clinical data among the study population.

**Parameters**	**All patients** **(*N* = 60)**	**Sustained hypertension** **(*N* = 29)**	**Normal ABPM** **(*N* = 31)**	***p*-value**
Age (years)	12.9 ± 3.7	12.1 ± 3.5	13.7 ± 3.7	0.76
Male, *N* (%)	38 (63)	16 (55)	22 (71)	0.21
Body weight (kg)	62.7 ± 23.8	63.8 ± 25.4	61.7 ± 22.6	0.73
Height (m)	1.54 ± 0.20	1.50 ± 0.21	1.58 ± 1.82	0.13
BMI (kg/m^2^)	25.5 ± 6.4	27.1 ± 6.3	23.9 ± 6.1	0.05
BMI z-score	1.80 ±1.57	2.32 ± 1.51	1.31 ± 1.49	0.01^*^
Obesity, *N* (%)	30 (50)	20 (69)	10 (32)	0.04^*^
Waist circumference (cm)	82 ± 17.7	86.7 ± 17.8	77.7 ± 16.7	0.048^*^
Waist to height ratio (cm/m)	53.3 ± 9.8	57.7 ± 8.5	49.2 ± 9.2	< 0.001^*^
Mean office SBP index	1.02 ± 0.07	1.04 ± 0.06	1.00 ± 0.07	0.09
Mean office DBP index	1.02 ± 0.07	1.03 ± 0.66	1.01 ± 0.76	0.38
Maximum office SBP index	1.07 ± 0.08	1.08 ± 0.06	1.05 ± 0.09	0.14
Maximum office DBP index	1.08 ± 0.07	1.09 ± 0.07	1.07 ± 0.75	0.35

Data presented as mean ± standard deviation.

* p ≤ 0.05.

### 3.2. ABPM parameters and phenotypes

The ABPM parameters between the sustained hypertension and normal ABPM groups are shown in Table 2 and the ABPM phenotypes between the obesity and non-obesity groups are shown in Table 3. Of 31 patients in the normal ABPM group, 12 patients had white coat hypertension and 19 patients had prehypertension, accounting for 20 and 31.7% of all high office blood pressure patients, respectively. In the sustained hypertension group (*N* = 29), seven patients had isolated daytime hypertension, 11 patients had isolated nocturnal hypertension and the remaining 11 patients had both daytime and nocturnal hypertension, accounting for 11.7, 18.3, and 18.3% of all high office blood pressure patients, respectively.

**Table 2:** Ambulatory blood pressure parameters between the sustained hypertension and normal ABPM groups.

**Parameters**	**All patients** **(*N* = 60)**	**Sustained hypertension** **(*N* = 29)**	**Normal ABPM** **(*N* = 31)**	***p*-value**
Daytime SBP index	0.96 ± 0.07	1 ± 0.07	0.92 ± 0.05	< 0.001^*^
Daytime DBP index^#^	0.87 (0.10)	0.88 (0.10)	0.85 (0.08)	0.021^*^
Nighttime SBP index	0.96 ± 0.08	1.01 ± 0.07	0.91 ± 0.05	< 0.001^*^
Nighttime DBP index^#^	0.91 (0.10)	0.92 (0.12)	0.89 (0.08)	0.203
24-h SBP index^#^	0.98 (0.09)	1.01 (0.06)	0.94 (0.07)	< 0.001^*^
24-h DBP index^#^	0.89 (0.09)	0.92 (0.09)	0.86 (0.08)	0.01^*^

Data presented as mean ± standard deviation.

# Median (IQR).

* p ≤ 0.05.

**Table 3:** Ambulatory blood pressure phenotypes between the obesity and non-obesity groups.

**Parameters**	**All patients** **(*N* = 60)**	**Obesity** **(*N* = 30)**	**Non-obesity** **(*N* = 30)**	***p*-value**
Normal ABPM group	31 (51.7)	10 (33.3)	21 (70)	
- White coat hypertension	12 (20)	3 (10)	9 (30)	0.053
- Pre-hypertension	19 (31.7)	7 (23.3)	12 (40)	0.165
Sustained hypertension group	29 (48.3)	20 (66.7)	9 (30)	
- Isolated daytime hypertension	7 (11.7)	6 (20)	1 (3.3)	0.044^*^
- Isolated nocturnal hypertension	11 (18.3)	7 (23.3)	4 (13.3)	0.317
- Both daytime and nocturnal hypertension	11 (18.3)	7 (23.3)	4 (13.3)	0.317

Data presented as N (% of total patients).

* p ≤ 0.05.

### 3.3. Parameters associated with sustained hypertension

Univariate analysis revealed that BMI z-score and WHtR were significantly associated with sustained hypertension as shown in Table 4. In addition, having obesity was 4.7 times (95% CI 1.571–13.866, *p* < 0.01) higher risk of sustained hypertension compared with those who did not have obesity while having WHtR ≥ 50 cm/m was 6.7 times (95% CI 2.004–22.041, *p* < 0.01) higher risk of sustained hypertension compared with those who had a WHtR < 50 cm/m. However, multivariate analysis revealed that WHtR was the only factor associated with sustained hypertension (OR 1.2; 95% CI 1.022–1.408, *p* = 0.026).

**Table 4:** Multivariate analysis of parameters associated with sustained hypertension.

**Parameters**	**Univariate analysis**			**Multivariate analysis**		
	**Exp (**β**)**	**95% CI**	***p*** **-value**	**Exp (**β**)**	**95% CI**	***p*** **-value**
**Demographic data**						
Age	0.875	0.754–1.017	0.081	0.890	0.764–1.038	0.139
Gender	1.986	0.684–5.769	0.207	1.586	0.515–4.880	0.422
**Anthropometric data**						
BMI	1.088	0.997–1.187	0.058			
BMI z-score	1.632	1.087–2.449	0.018^*^	0.691	0.296–1.611	0.392
Waist circumference	1.032	0.999–1.066	0.055			
Waist to height ratio	1.123	1.044–1.209	0.002^*^	1.2	1.022–1.408	0.026^*^
**Office BP indices (for each 0.1 increase)**						
Mean office SBP	1.999	0.897–4.455	0.090			
Mean office DBP	1.395	0.674–2.887	0.369			
Maximum office SBP	1.703	0.842–3.444	0.138			
Maximum office DBP	1.426	0.680–2.991	0.348			

BMI, body mass index; BP, blood pressure; SBP, systolic blood pressure; DBP, diastolic blood pressure.

* p ≤ 0.05.

### 3.4. Performance of BMI z-score and WHtR

**Figure 1:**
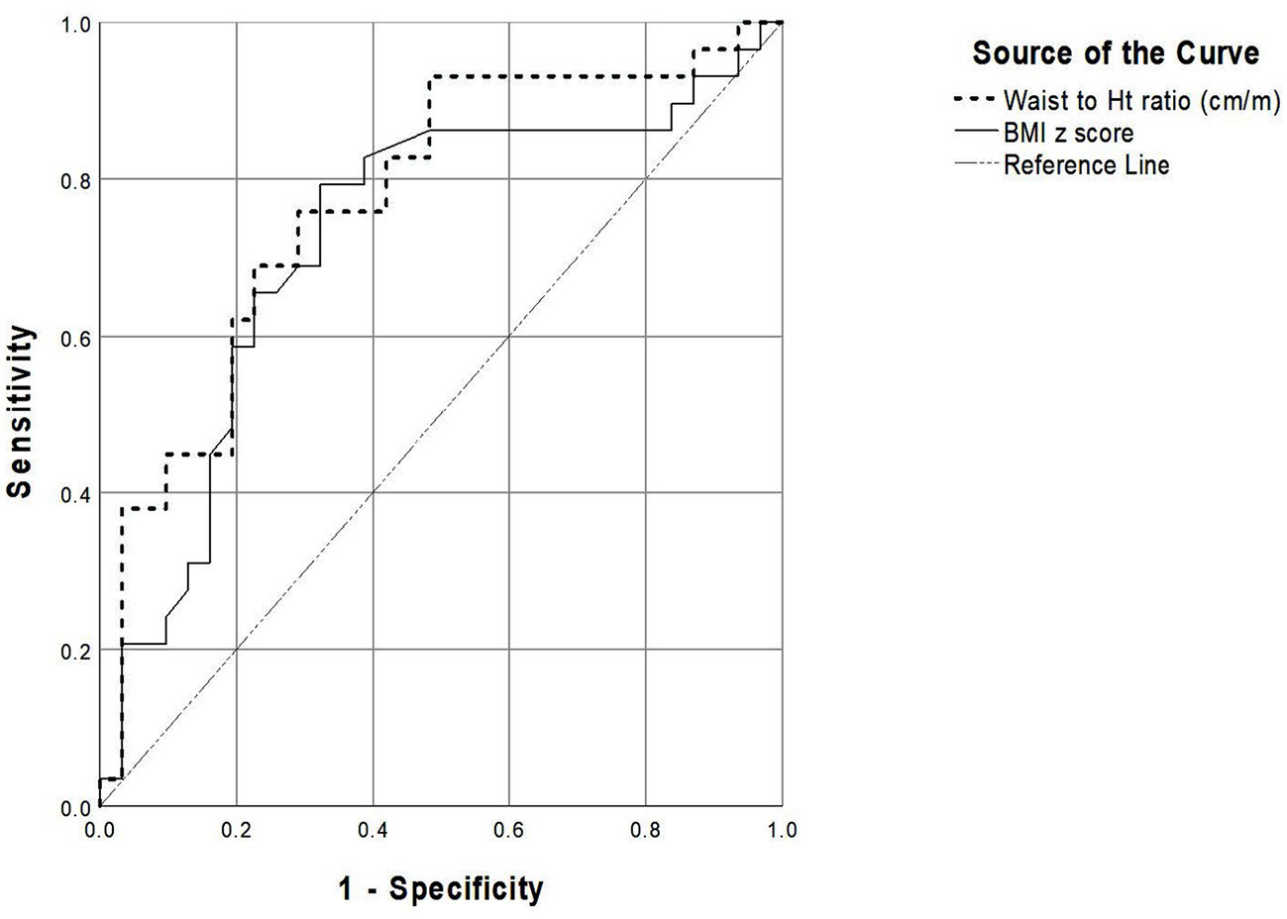
The receiver-operating characteristic curve representing performance of the parameters for detecting sustained hypertension.

For the diagnosis of sustained hypertension, the ROC analysis revealed that WHtR had a greater area under the curve (AUC) than that of BMI z-score (0.772 vs. 0.723, respectively). WHtR ≥ 50 cm/m had a sensitivity of 82.8% and a specificity of 58.1% whereas BMI z-score > 2 had a sensitivity of 69% and a specificity of 67.7% to detect sustained hypertension (Figure 1, Table 5).

**Table 5:** Adiposity parameters for detecting sustained hypertension.

**Indicators**	**Cut-off**	**Sensitivity**	**Specificity**	**AUC**	**95% CI**	***p*-value**
BMI z-score	1.0	86.2	38.7	0.723	0.588–0.858	0.003^*^
	1.5	82.8	51.6			
	2.0	69	67.7			
WHtR (cm/m)	45	93.1	29	0.772	0.651–0.893	< 0.001^*^
	50	82.8	58.1			
	55	62.1	80.6			

AUC, area under the curve; 95% CI, 95% confidence interval.

* p ≤ 0.05.

## 4. Discussion

Among children and adolescents suspected to have hypertension due to the detection of persistently high office blood pressure, the present study revealed that 48.3% had sustained hypertension. This was consistent with the previous studies reporting the prevalences of sustained hypertension ranging from 20 to 54% (37–39). The present study also showed that WHtR tended to outperform BMI z-score for the prediction of sustained hypertension.

The associations between ABPM parameters and obesity have been well-described. The previous studies reported that white coat hypertension was seen in 10–30% of pediatric patients with obesity (31, 40, 41). These results were consistent with the present study that white coat hypertension was seen in 10% of patients. For nocturnal hypertension, the prevalences ranged from 17 to 23% in pediatric patients with obesity (41, 42) compared to the prevalence of 46.6% in the present study. It was postulated that patients with obesity had high nighttime blood pressure than daytime blood pressure due to poor sleep quality caused by snoring, altered function of the autonomic nervous system, or an impaired ability to excrete sodium (40).

In the present study, not only BMI z-score but also was WHtR significantly higher in the sustained hypertension group than the normal ABPM group. After adjusting with age, gender, and BMI z-score, WHtR was found to be the only independent parameter associated with sustained hypertension. In addition, WHtR at the cut-off point > 0.5 showed a good sensitivity of 82.8% while BMI z-score at the cut-off point of >2 (WHO criteria for obesity) showed a sensitivity of 69% to detect sustained hypertension. Therefore, WHtR tended to be a slightly better parameter for predicting sustained hypertension than BMI z-score.

While WHtR is a parameter representing abdominal obesity, BMI is a parameter representing total body mass. As BMI cannot distinguish between fat and fat-free mass, an elevated BMI may not entirely reflect adiposity accumulation (43). On the other hand, WHtR is related to the amount of intra-abdominal visceral fat, which is more closely related to cardiovascular risk compared with total body mass represented by BMI (21, 44). For implementing across various age groups, BMI varies significantly according to child growth and pubertal development, so it must be stated as a z-score to age and gender. On the contrary, WHtR varies slightly by age and gender and does not need to be stated as a z-score because waist circumference and children's height increase continuously as they age in the same boundary value (25, 44). As a result, WHtR becomes a simpler index to calculate. Not only for sustained hypertension, WHtR also was reported to perform well to predict metabolic syndrome in a national survey of Thai adolescents (45).

Although many studies (10, 21, 26–29) had shown a strong association between WHtR and hypertension, some other studies showed that BMI z-score is slightly superior to or equal to WHtR (14, 18, 30, 46). The discordant results of those studies from the present study could be due to variation in the study designs. Those studies used different definitions of hypertension and most used one- to two-visits of office blood pressure measurements, or used persistently elevated office blood pressure. However, the present study categorized hypertension according to the results of ABPM as recommended by the current pediatric guidelines (3).

The available data regarding the performance of WHtR and the results of ABPM in children and adolescents with persistently high office blood pressure are limited. To our knowledge, this is the largest study that analyzed the performance of WHtR in predicting sustained hypertension by using ABPM. Nonetheless, the present study had some limitations. Firstly, auscultatory blood pressure was not performed for office blood pressure measurement. The other metabolic abnormalities associated with cardiovascular risks such as dyslipidemia and insulin resistance had not been collected. Data regarding the end-organ damages such as left ventricular mass index which is well-known and associated with sustained hypertension were unavailable. Lastly, the number of patients included in the present study was small. A larger study needs to be conducted to explore the performance of WHtR in predicting the risk of sustained hypertension among children with persistently high office blood pressure.

In conclusion, sustained hypertension was detected in 48.3% of the patients with persistently high office blood pressure. Apart from being a more user-friendly metric, WHtR tended to outperform BMI z-score in predicting sustained hypertension confirmed by ABPM.

## Data availability statement

The raw data supporting the conclusions of this article will be made available by the authors, without undue reservation.

## Ethics statement

The studies involving human participants were reviewed and approved by the Ethics Committee for Human Research of Ramathibodi Hospital (MURA 2022/327). Written informed consent from the participants' legal guardian/next of kin was not required to participate in this study in accordance with the national legislation and the institutional requirements.

## Author contributions

NN, AS, KP, WP, SC, PS, and KT designed the study. NN, AS, KP, and WP performed the study. NN, AS, and KP drafted and revised the manuscript. All authors approved the final version of the manuscript.
